# Separated-pair approximation and separated-pair pair-density functional theory†

**DOI:** 10.1039/c5sc03321g

**Published:** 2015-12-16

**Authors:** Samuel O. Odoh, Giovanni Li Manni, Rebecca K. Carlson, Donald G. Truhlar, Laura Gagliardi

**Affiliations:** a Department of Chemistry, Chemical Theory Center, Supercomputing Institute, University of Minnesota 207 Pleasant Street SE Minneapolis MN 55455-0431 USA gagliardi@umn.edu truhlar@umn.edu; b Max-Planck Institut für Festkörperforshung Heisenbergstraße 1 70569 Stuttgart Germany

## Abstract

Multi-configuration pair-density functional theory (MC-PDFT) has proved to be a powerful way to combine the capabilities of multi-configuration self-consistent-field theory to represent the an electronic wave function with a highly efficient way to include dynamic correlation energy by density functional theory. All applications reported previously involved complete active space self-consistent-field (CASSCF) theory for the reference wave function. For treating large systems efficiently, it is necessary to ask whether good accuracy is retained when using less complete configuration interaction spaces. To answer this question, we present here calculations employing MC-PDFT with the separated pair (SP) approximation, which is a special case (defined in this article) of generalized active space self-consistent-field (GASSCF) theory in which no more than two orbitals are included in any GAS subspace and in which inter-subspace excitations are excluded. This special case of MC-PDFT will be called SP-PDFT. In SP-PDFT, the electronic kinetic energy and the classical Coulomb energy, the electronic density and its gradient, and the on-top pair density and its gradient are obtained from an SP approximation wave function; the electronic energy is then calculated from the first two of these quantities and an on-top density functional of the last four. The accuracy of the SP-PDFT method for predicting the structural properties and bond dissociation energies of twelve diatomic molecules and two triatomic molecules is compared to the SP approximation itself and to CASSCF, MC-PDFT based on CASSCF, CASSCF followed by second order perturbation theory (CASPT2), and Kohn–Sham density functional theory with the PBE exchange–correlation potential. We show that SP-PDFT reproduces the accuracy of MC-PDFT based on the corresponding CASSCF wave function for predicting C–H bond dissociation energies, the reaction barriers of pericyclic reactions and the properties of open-shell singlet systems, all at only a small fraction of the computational cost.

## Abstract 2

Here we present the separated-pair approximation for wave function theory and show that it performs almost as well as the more demanding complete active space approximation. We show that the combination of an SP wave function with an on-top density functional yields comparable accuracy to CASPT2 at a small fraction of the cost.

## Graphical abstract

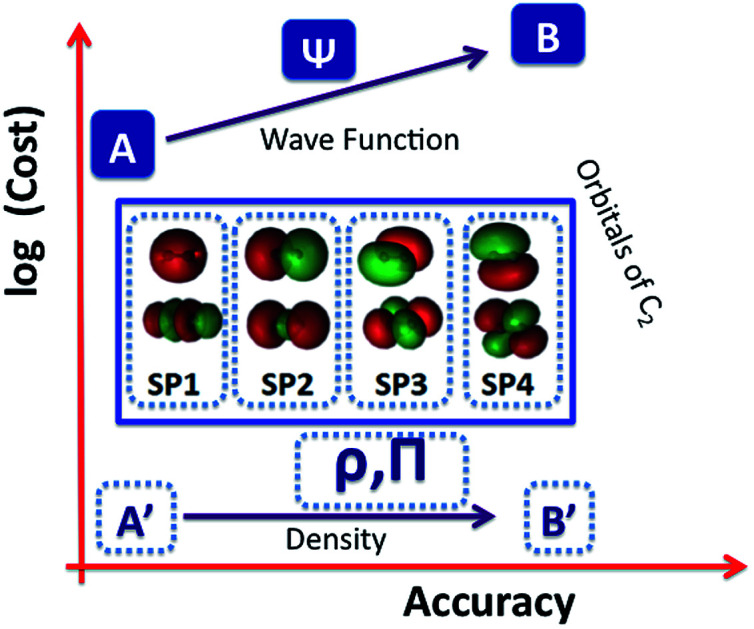

## Introduction

There is strong interest in the development of quantum chemical methods for accurately treating large systems with inherently multiconfigurational electronic structures at affordable computational cost.^1^ Such systems are also called multireference systems or strongly correlated systems, and they are usually treated, at least as a first-order approximation, by multiconfigurational self-consistent field (MCSCF) methods.^2^ This approach includes static electron correlation that would be neglected if a single electronic configuration were employed. In MCSCF methods, one simultaneously variationally optimizes all the orbitals and the coefficients of the various configurations in a configuration interaction (CI) expansion of the electronic wave function. There are several possible ways to select the configurations that are included. In the complete active space self-consistent field (CASSCF) method, a full configuration interaction (FCI) expansion of the wave function is constructed over an active space of *n* electrons in *N* orbitals, with other orbitals double occupied (inactive) or vacant.^3^ The size of the FCI expansion grows exponentially as the active space is enlarged, such that an active space with *n* = 18 and *N* = 18, labeled as CAS(18,18), is already at the limit of what is affordable. For medium-to-large systems, the active space limit, CAS(18,18), is typically not large enough to describe bond-breaking, electronic excitations, and other chemical properties in a balanced fashion. Thus well-balanced CASSCF calculations are in practice limited to the study of small-to-medium systems.

Generally, most of the configurations in the FCI expansion of the active space in CASSCF computations make only small contributions to the total wave function. As a result, Ruedenberg and coworkers suggested that these configuration state functions (CSFs) are “deadwood” that can be excluded without significantly affecting the accuracy of the results.^4^ The generalized active space (GAS),^5,6^ restricted active space (RAS),^7,8^ occupation restricted multiple active spaces (ORMAS)^9^ and Split-GAS^10,11^ approaches are some of the frameworks that attempt to remove deadwood CSFs by partitioning the active space into subspaces. We have previously shown that active spaces larger than the CAS(18,18) limit can be attained with the generalized active space self-consistent-field theory, GASSCF.^6,10,11^

These MCSCF-type wave functions (CASSCF, GASSCF, *etc.*) can recover static correlation effects well, but are impractically slowly convergent (with respect to active space size) for the dynamic correlation energy, which is necessary for chemically accurate energetic calculations. For higher accuracy they can be used as zeroth-order reference functions in post-SCF perturbative, multireference coupled-cluster (CC), or multireference configuration interaction (CI) calculations to obtain a good approximation to the dynamic correlation energy. CASPT2 is a popular example that applies second-order perturbation theory to a CASSCF zero-order wave function.^12,13^ Such approaches, while capable of high accuracy,^8,14^ are however not suited for studying large systems because their computational costs rise rapidly with system size.

We have recently proposed an approach for treating strongly correlated systems at much lower computational costs than CASPT2, by combining CASSCF with density functional theory (DFT). This approach is called multiconfiguration pair-density functional theory (MC-PDFT).^15,16^ It may be considered to be a multiconfigurational analog of Kohn–Sham^16^ density functional theory^16,17^ (KS-DFT). In KS-DFT, the energy is computed as the kinetic energy and classical Coulomb energy of a Slater determinant (which is a single-configuration reference wave function) and a one-electron integral over an exchange–correlation functional of the one-electron density of the Slater determinant. The classical Coulomb energy includes the nuclear attraction of the electrons, the classical interelectronic repulsion of the electronic charge density, and the nuclear repulsion. The exchange–correlation density functional includes electron exchange, electron correlation, and the difference between the exact kinetic energy and that computed from the Slater determinant. The exact exchange–correlation density functional is unknown, so one uses approximations. In MC-PDFT, the energy is computed as the kinetic energy and classical Coulomb energy of an MCSCF reference wave function and a one-electron integral over an on-top density functional of the one-electron density and the on-top pair density of the reference wave function. The on-top density functional includes electron exchange, electron correlation, and the difference between the exact kinetic energy and that computed from the reference wave function. The MC-PDFT energy may be written as1
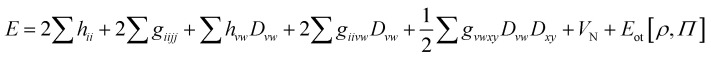
where orbital indices refer to the spatial molecular orbitals, *i* and *j* are the doubly occupied inactive orbitals, *v*, *w*, *x*, and *y* are the active orbitals, *h*_*vw*_ and *g*_*pqrs*_ respectively one-electron and two-electron integrals, *D*_*pq*_ is the one-electron reduced density matrix, *V*_N_ is the nuclear repulsion, and *E*_ot_[*ρ*,*Π*] is an on-top density functional of the total density, *ρ*, and the on-top pair density, *Π*. Functional expressions for *E*_ot_[*ρ*,*Π*] when using *ρ* and *Π* obtained from an MCSCF solution have been provided in ref. 15 and 18

KS-DFT is usually applied full self-consistently; that is, the exchange–correlation functional is included during the SCF step. MC-PDFT can also in principle be applied fully self-consistently, but in all work reported so far and in the present article, we carry out the MCSCF calculation by CASSCF without the on-top density functional, and then calculate the final energy post-SCF from eqn (1). In this post-SCF mode, MC-PDFT is like the perturbation theory, multireference CC, and multireference CI wave function methods in that it attempts to use an MCSCF method to obtain a balanced reference wave function in an SCF step and to calculate an accurate energy in a post-SCF step. However, in the case of MC-PDFT, the cost of the post-SCF density functional step is negligible (if coded efficiently) compared to the cost of the SCF step, whereas in the wave function methods like CASPT2, the post-SCF step is more expensive than the SCF step. The cost of the SCF step though is still prohibitive for large systems if one uses CASSCF as the MCSCF method. In the present article we test whether MC-PDFT can yield accurate results when based on a GASSCF wave function. In particular, we present a systematic way to choose the active space in GASSCF theory. This new way of choosing the active space is called the separated-pair (SP) approximation. The method is intermediate between generalized valence bond (GVB) theory and complete active space self-consistent-field (CASSCF) theory. We then use SP and CASSCF as reference wave functions for MC-PDFT. The MC-PDFT method based on a CASSCF and a SP reference wave function will be labeled as CAS-PDFT and SP-PDFT, respectively, when it is desired to distinguish the kind of MCSCF wave function being used as the reference.

The next section presents the relevant theory and defines the separated pair (SP) approximation. We then provide computational details, test sets, results, and discussion.

## Theory

### On-top density functionals

We have previously presented a prescription for translating existing exchange–correlation functionals of KS-DFT to on-top functionals.^15^ As an example, tPBE is an on-top pair density functional developed by translating the PBE functional;^15^ tPBE is a function of the electron density, its gradient, and the on-top pair density. We have also described a “fully” translated functional called ftPBE that also depends on the gradient of the on-top pair density.^18,19^

### Separated pair approximation

The first step in building a GASSCF wave function is to choose the number *m* of GAS subspaces and the number and type of orbitals in each GAS subspace. Note that not only in eqn (1) but also in the whole rest of the article, when we talk about orbitals, we are referring to spatial orbitals, not spin-orbitals. We use the notation GAS-*m*(*n*,*N*) for *n* electrons in *N* orbitals divided into *m* subspaces. But this is not a complete specification; in addition, for each irreducible representation, one specifies the accumulated minimum and maximum electron occupations after each GAS subspace is added. For a GAS-*m*(*n*,*N*) calculation, the number of electrons in each space, the number and nature of orbitals in each space, and the number of inter-subspace excitations can significantly affect the number of CSFs in the CI expansion, and – by extension – the quality of the results obtained. A GAS wave function includes all configurations that can be defined within the restrictions imposed by the accumulated minimum and maximum electron occupations and by the restriction, if any, on inter-subspace excitations. The effects of these specifications on the computed properties of various molecules have been previously reported.^5,6,11,20^

In the present work, we only use GAS subspaces in which each subspace contains at most two orbitals, and interspace excitations are not allowed. A GASSCF calculation with these restrictions will be called the separated pair (SP) approximation, and when the number of subspaces is *m*, it will be abbreviated SP-*m*. If each subspace contains two electrons in two orbitals, this would be specified in the language of GASSCF as GAS-*m*(2*m*,2*m*) with the additional specification that no inter-subspace excitations are allowed. For singlet systems with an even number of electrons, we typically do have two electrons in two orbitals in each subspace, and the two orbitals in a given subspace are usually a bonding orbital and the corresponding antibonding orbital. This is reminiscent of the generalized valence bond perfect pairing (GVB-PP) algorithm,^21^ but it is more general. The GVB-PP approximation has subspaces of two electrons in two orbitals coupled to a singlet; this involves two or three configurations, depending on symmetry. In the SP approximation, when there are two electrons in two orbitals, they may be coupled into either a singlet or a triplet, and the various triplet pairs may be coupled in all possible ways to obtain CSFs with the desired overall spin symmetry of the system (for example, if the overall wave function is a singlet, one may have CSFs where four of the pairs are triplets and all the others are singlets, and the four triplet pairs may be coupled to each other in a variety of ways to obtain an overall singlet); thus the SP approximation involves more possible configurations than does the GVB-PP approximation. Nevertheless, the SP approximation greatly reduces the number of CSFs in the CI expansion as compared to CASSCF. It is also important to note that we carry out a FCI expansion in each GAS subspace. This is because we allow both singles and double excitations in each subspace containing just two orbitals. The SP approximation is more similar to the generalized valence bond restricted pairing (GVB-RP) approximation^22^ than to GVB-PP. A key advantage of SP and GVB-RP is that, unlike GVB-PP, they allow dissociation to high-spin fragments.^21,22^

In the SP approximation, every GAS subspace contains one electron in one or two orbitals or two or three electrons in two orbitals, depending on the system. Intersubspace excitations are always excluded. For closed-shell systems (and for open-shell singlets that can be made by breaking a bond in a closed-shell system) an SP-*m* approximation always corresponds to GAS-*m*(2*m*,2*m*). But the value of *m* depends on which pairs are included in the active space and which are treated as inactive (doubly occupied in all CSFs), and that is an individual choice. For example, we treat the molecular orbitals with parentage in the 2s atomic orbitals as active for C_2_ but inactive for N_2_, O_2_, and F_2_. Moss and coworkers,^23^ in their GVB-CI calculations on O_2_, also removed the fully occupied 1σ_g_, 1σ_u_, 2σ_g_ and 2σ_u_ molecular orbitals from the active space. For F_2_, we also treat the molecular orbitals with parentage in the 2p_*x*_ and 2p_*y*_ orbitals as inactive.

**Fig. 1:**
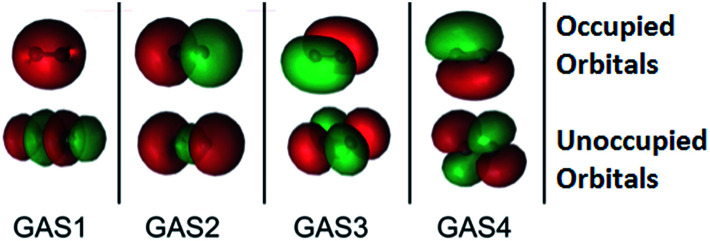
The four GAS subspaces used in the SP-4 approximation for the carbon dimer, C_2_. In this scheme, the 2s, 2p_*z*_, 2p_*x*_, and 2p_*y*_ atomic orbitals form σ_g_, σ_u_, π(p_*x*_), and π(p_*y*_) (which are bonding or in the case of 2σ_u_, GAS2, antibonding) orbitals respectively as well as their antibonding (or in the case of 2σ_u_, GAS2, bonding) counterparts. These pairs are shown from left to right. The orbitals with an occupation close to two are placed at the *top*, while those that are nearly empty are placed at the *bottom*. Two electrons are placed in each GAS subspace. Intra-space excitations (up to double excitations) between a bonding orbital and its antibonding pair are allowed. Inter-subspace excitations between GAS subspaces are not allowed.

The SP approximation we used for the carbon dimer, C_2_, is shown in Fig. 1. This molecule has a closed-shell singlet ground state, and the orbitals shown in Fig. 1 correspond formally (at equilibrium) to a double bond and a ground state configuration of 2σ_g^2^_, 2σ_u^2^_, 1π_ux^2^_, 1π_uy^2^_. This corresponds to a double bond as the occupied 2σ_u_ orbital is actually of antibonding character. Within *C*_1_ symmetry, there are 150 CSFs in this reference for the closed-shell singlet, as compared to 1764 CSFs in the analogous CAS(8,8) reference (the analogous GVB-PP wave function would have only 16 CSFs). We note that the SP-4 reference correctly dissociates to two high-spin (^3^P) carbon atoms, just like the CAS(8,8) reference.

**Fig. 2:**
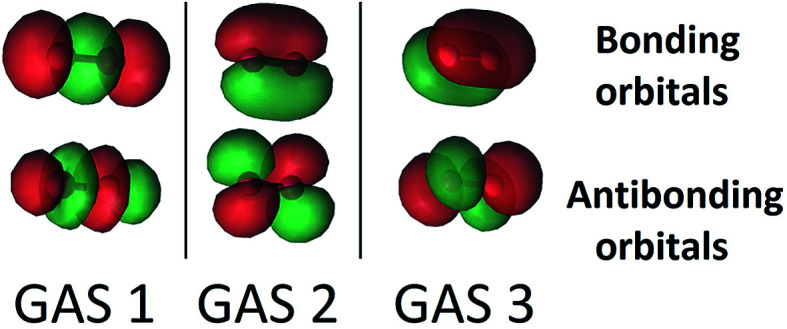
The three GAS subspaces used in SP calculations on triplet dioxygen, O_2_. In this scheme, the 2p_*z*_ atomic orbitals form 3σ and 3σ* orbitals, and the 2p_*x*_ and 2p_*y*_ atomic orbitals form bonding π(p_*x*_), and π(p_*y*_) orbitals and correlating antibonding π*(p_*x*_), and π*(p_*y*_) orbitals. These are shown from left to right. GAS 1 contains two electrons while GAS 2 and GAS 3 each contain 3 electrons. Inter-subspace excitations between GAS spaces are not allowed.

The SP scheme for open-shell systems depends on the type of open-shell character. The SP-3 approximation that we used for O_2_ is shown in Fig. 2. O_2_ differs from C_2_ in that the σ bonding combination of 2p orbitals lies higher in energy than the π bonding combination for C_2_ but lower for O_2_. In O_2_, as already mentioned, the 2σ and 2σ* molecular orbitals (which are predominantly formed from the 2s atomic orbitals) are kept inactive. Therefore the SP-3 approximation that we used for O_2_ has GAS1 containing two electrons in the 3σ_g_ and 3σ_u_ orbitals (which are predominantly formed from the 2p_*z*_ atomic orbitals), GAS2 containing three electrons in the 1π(p_*x*_) and 1π*(p_*x*_) orbitals, and GAS3 containing three electrons in the 1π(p_*y*_) and 1π*(p_*y*_) orbitals. This is a GAS-3(8,6) reference. It contains 20 CSFs in comparison to 378 CSFs for the full valence CAS(12,8) and 105 CSFs for CAS(8,6). This GAS-3(8,6) reference also correctly dissociates into two ^3^P oxygen atoms. The SP approximations we used for SO and S_2_ are isoelectronic to that for O_2_.

For the Cr dimer, Cr_2_, we calculated the potential energy curve with an SP-6 approximation, equivalent to GAS-6(12,12), with the twelve valence orbitals coupled in six GAS subspaces and two active electrons in each GAS subspace. Within *D*_2h_ symmetry, there are 1516 CSFs in the SP-6 CI expansion, as compared to 28 784 CSFs in the analogous CAS(12,12) CI expansion. The SP-6 approximation is sufficiently complete that the dimer correctly dissociates to two high-spin (^7^S Cr) atoms.

For methylene triplet or methylene open-shell singlet, a full valence CAS is (6,6). We can think of CH_2_ as derived from methane by dissociating two C–H bonds, and the antibonding orbitals associated with those bonds have left with the hydrogens. Thus these systems each have two singly occupied orbitals, which are taken as their own GAS subspaces with one electron in one orbital in each. In addition, they have two GAS spaces that each have two electrons in two orbitals. Thus the separated pair approximation we use is SP-4, which is shorthand in this case for GAS-4(6,6).

There are two important points to note. First, the SP approximation allows one to design GAS subspaces that contain only the bonding and antibonding orbitals necessary to describe a particular process. For example to compute the C–H dissociation energies of acetylene, ethylene and ethane, we included only orbitals relevant to C–H bonding in the SP active space. This formally leads to a SP-3 active space for both acetylene and ethynyl, an SP-4 active space for both ethylene and vinyl, and an SP-6 active space for both ethane and ethyl. We illustrate this feature with several examples. For the ethyl radical, a full valence CAS space would be (13,13) with seven bonds. We think of this as derived from ethane by removing a hydrogen atom, and the antibonding orbital accompanies it. Constructing GAS subspaces with the same logic as explained above for methylene then yields an SP-7 approximation that is equivalent to GAS-7(13,13). However, when we study C–H bond dissociation in this paper, we treat the C–C bonding orbital as inactive and use an SP-6 approximation corresponding to GAS-6(11,11). Ethynyl has a full-valence CAS of size (9,9). Since we are interested in C–H bond dissociation, we made the four electrons in π and π* orbitals inactive, which yields an SP-3 approximation equivalent to a GAS-3(5,5) reference. Vinyl has a full valence CAS of size (11,11). Since we are interested in C–H bond dissociation in, and because we are interested in seeing the effect of aggressively reducing the size of the active space, we removed the four electrons in the C=C bond and the associated σ, σ*, π, and π* orbitals from the active space, which yields an SP-4 approximation, equivalent to GAS-4(7,7).

Second, the SP-1 approximation is equivalent to CASSCF(2,2), a case which applies to lithium hydride (LiH), as an example. In addition, as we are performing a full CI for each subspace, SP and SP-PDFT are size consistent in so far as the active space is chosen correctly. For all other molecules, the SP approximation used here involves an active pair for all or some of the bonds, as specified in each case. Nonbonding valence orbitals and core orbitals are always doubly occupied.

## Computational details

### Basis sets

The aug-cc-pVTZ basis set is used to describe all the H, Li, B, C, N, O, F and S atoms in the molecules studied in this work.^24^ For the Cr dimer we used the ANO-RCC basis set^25^ containing [21s15p10d6f4g2h] primitive functions contracted to (10s10p8d6f4g2h).

### Symmetry

For the Cr dimer, a *D*_2h_ point group was adopted. All other calculations in this work were carried out without symmetry. This is because the method is designed for large molecules that usually have no symmetry so we want to test it in that context.

### CASSCF calculations

We used full valence active spaces in CASSCF calculations on all the molecules studied in this work. The exceptions are ozone, for which we used CAS(12,9), α-3-didehydrotoluene and 1,4-didehydrobenzene, for which we used CAS(8,8), and the compounds involved in pericyclic reactions for which we included only the subset of π, π*, σ, and σ* orbitals of the carbon ring systems that are transformed during the reaction. The full details of the active space used for each compound are given in the ESI.†

### CASPT2 and CAS-PDFT

To include dynamic correlation, the CASSCF solutions are used as references in MC-PDFT and CASPT2 calculations. For MC-PDFT, we used the CAS-tPBE and CAS-ftPBE functionals.^15^ These are our translated and fully translated functionals that use CASSCF solutions as references. For CASPT2, an empirical ionization-potential – electron-affinity (IPEA) shift of 0.25 atomic units (6.80 eV) is added to improve agreement with experiment.^26^ To illustrate the dependence of CASPT2 on this empirical parameter and to allow for a more standard comparison with MC-PDFT, we performed analogous calculations without the IPEA shift. These calculations are labeled as CASPT2-0. For the Cr dimer we also employed an IPEA value of 0.45 atomic units, as suggested for this specific system in previous work.^8^ All CASPT2 and CASPT2-0 computations used a standard imaginary shift of 0.2 atomic units (5.44 eV) to prevent intruder states.^27^

### SP calculations

As in CASSCF and GASSCF in general, the CI coefficients are optimized *via* a Direct-CI procedure^28^ while the orbital parameters are optimized through the Super-CI approach.^29^ Intra-space rotations (inactive–inactive, virtual–virtual, gas1–gas1, gas2–gas2, …) are redundant and are not included in the optimization step; only inter-subspace rotations are included in the orbital optimization procedure.

### SP-PDFT calculations

SP-PDFT calculations are just like CAS-PDFT calculations, except that the reference wave function is a separated pairs approximation.

### KS-DFT calculations

The results of calculations with CASPT2, CASPT2-0, CAS-tPBE, CAS-ftPBE, SP-tPBE and SP-ftPBE are compared with those obtained from KS-DFT calculations with the PBE^30^ exchange–correlation functional.

### Geometries

We used the experimental geometries of acetylene and ethylene as well as those of the ethynyl and vinyl radicals.^31^ We optimized the structures of ethane and the ethyl radical by M06-2X^32^/6-31G(d). For the pericyclic reactions, the geometries and zero point energies of the reactants and transition states were obtained at the B3LYP/6-31G(d) level by Houk and coworkers.^33^ The geometries of methylene and ozone were optimized by scanning the bond lengths at various bond angles. The geometries of planar and twisted ethylene were obtained with the MR-CISD/SA-3-RDP/aug-cc-pVTZ method by Lischka and coworkers.^34^ For α-3-didehydrotoluene and 1,4-didehydrobenzene, we use geometries optimized at the M06-2X^32^/6-31G(d) level while using unrestricted Kohn–Sham DFT (abbreviated as UDFT).

### Bond energies and atomization energies

All bond energies and atomization energies in this paper are potential energy differences excluding vibrational energies. Usually these are obtained from the literature, but for CH_2_ the thermal correction to the enthalpy at 298 K obtained by KS-PBE/aug-cc-pVTZ is added to the empirical Δ*H*_298_ of CH_2_. The frequency component of this correction was scaled using the scaling factor obtained from ref. 35 for this model chemistry.

### Software

All the CASSCF, CASPT2, CASPT2-0, SP, and SP-PDFT calculations in this work were carried out with a locally modified version of the *Molcas* 7.9 program suite.^36^ All KS-DFT calculations were carried out with the *Gaussian 09* program.^37^

### Systems studied

In order to provide a broad test of the performance of SP-PDFT, we have computed the structural properties and bond energies of twelve diatomic molecules (LiH, HF, B_2_, C_2_, CO, S_2_, SO, NH, N_2_, O_2_, F_2_, and Cr_2_) and two triatomic molecules (CH_2_ and O_3_). We also studied C–H bond dissociation in three prototypical organic compounds (acetylene, ethylene and ethane) and the barrier heights of five pericyclic reactions. The pericyclic reactions are the electrocyclic ring opening of cyclobutene, the ring closing of *cis*-1,3,5-hexatriene and *ortho*-xylylene, and the sigmatropic shift reactions of 1,3-pentadiene and 1,3-cyclopentadiene. Finally, we examined the performance of SP-PDFT for describing the properties of open-shell singlet (OSS) systems, specifically the relative energies of planar and twisted ethylene, and the singlet–triplet separations in α-3-didehydrotoluene and 1,4-didehydrobenzene.

## Results and discussion

### Diatomic molecules

The ability of an electronic structure method to provide potential energy surfaces or potential energy curves that accurately describe the formation and cleavage of chemical bonds is a very important test of its capabilities. This task is challenging for methods based on a single-configuration reference state; for example, coupled cluster theory with full inclusion of single, double, and triple excitations (CCSDT) fails to properly describe the dissociation of N_2_ into two N atoms.^38^ The spectroscopic constants (the equilibrium distances, *R*_e_, and dissociation energies, *D*_e_) of diatomic molecules have been computed with many theoretical methods (see ref. 39 for examples), and they are good test cases to compare the results obtained from SP-PDFT to those obtained with CAS-PDFT and CASPT2 as well as to accurate experimental data.

### Equilibrium bond distances of diatomic molecules

**Fig. 3:**
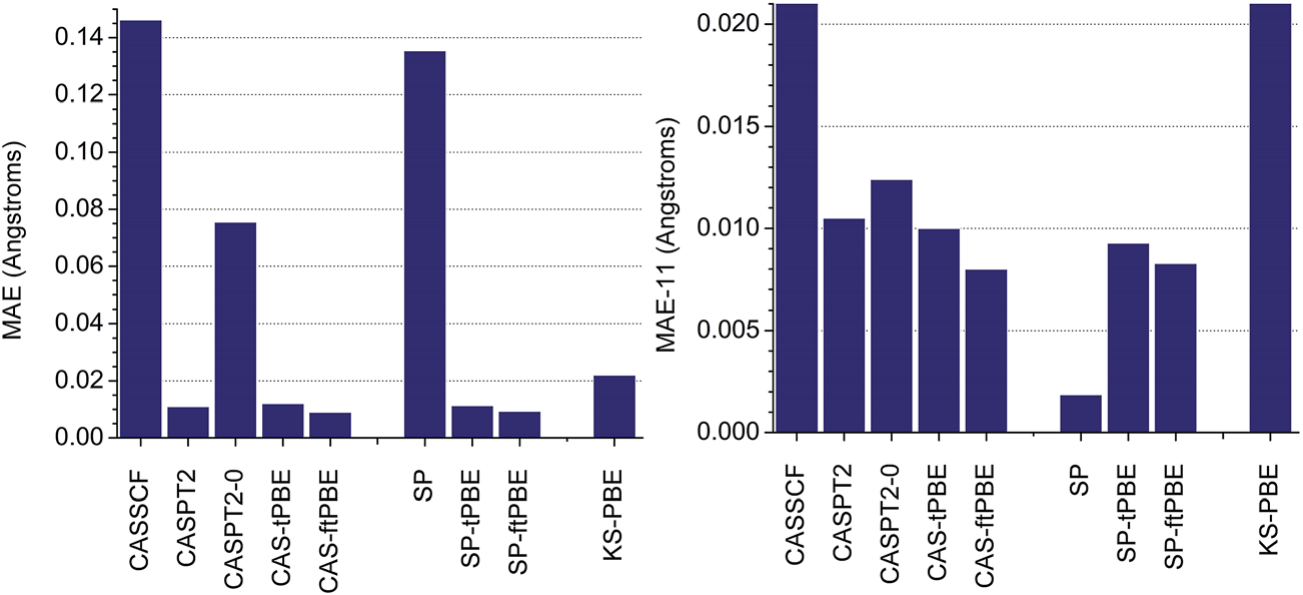
Mean absolute errors (MAE) with respect to experimental values of the calculated bond distances of eleven main-group diatomic molecules and the chromium dimer, Cr_2_, obtained with several computational approaches (*left*). The MAE obtained without the results for Cr_2_ (labeled as MAE-11) is shown on the *right*. The theoretical methods are grouped into three classes. The first are based on CASSCF (CASSCF, CASPT2, CASPT2-0, CAS-tPBE, CAS-ftPBE). The second are based on the SP approximation (SP, SP-tPBE, SP-ftPBE). The third is Kohn–Sham DFT with the PBE exchange–correlation functional. All experimental data were obtained from ref. 40.

In Fig. 3, we show the performance of SP-PDFT and other methods for predicting the equilibrium bond distances of eleven diverse main-group diatomic molecules, LiH, HF, B_2_, C_2_, CO, S_2_, SO, NH, N_2_, O_2_, and F_2_, and one transition-metal diatomic molecule, Cr_2_. The dominant configurations in the CASSCF wave functions when using full-valence complete active spaces have percentage weights of 98.0, 99.9, 78.5, 70.9, 94.3, 94.8, 95.0, 98.3, 92.8, 94.0, 93.2, and 44.3, respectively. Since molecules in which the dominant configuration has a weight of less than or equal to 95% are usually considered to be multireference, we see that nine of the twelve molecules are multireference ones, the Cr_2_ case being the one least dominated by a single configuration, followed by B_2_ and C_2_.

It has previously been recognized that CASSCF solutions generally lead to equilibrium bond lengths that are too long,^13^ and our results are consistent with this. CASSCF has a mean absolute error (MAE) of 0.146 Å when compared to experimental data. This statistic is however dominated by the result obtained for Cr_2_, for which CASSCF overestimates the equilibrium bond length by 1.52 Å. Without the results obtained for Cr_2_, the MAE of CASSCF (labeled as MAE-11) is 0.021 Å. This is similar to previous results.^13^ The MAE-11 of KS-PBE (0.022 Å) is similar to that of CASSCF. However, we find that the CAS-tPBE and CAS-ftPBE methods reduce the MAEs of both CASSCF and KS-PBE by in excess of 50%. As shown in Fig. 3, CAS-tPBE (MAE of 0.012 Å and MAE-11 of 0.010 Å) and CAS-ftPBE (MAE of 0.009 Å and MAE of 0.008 Å) perform as well as the much more expensive CASPT2 method (MAE of 0.011 Å and MAE-11 of 0.011 Å), with CAS-ftPBE being the best of the three approaches for bond distances. Without the IPEA shift, CASPT2 (labeled as CASPT2-0), performs poorly for Cr_2_, resulting in a MAE of 0.076 Å. Even without the data for Cr_2_, CASPT2-0 (MAE-11 of 0.012 Å) is not as good as CAS-tPBE and CAS-ftPBE.

Examination of Table S1† (tables and figures numbered with the prefix S are in the ESI†) indicates that CASPT2 and CASPT2-0 significantly outperform CAS-PDFT and SP-PDFT only for the bond length of the fluorine molecular dimer, F_2_. CAS-tPBE and CAS-ftPBE underestimate the bond-length of F_2_ by 0.021–0.023 Å, while CASPT2 and CASPT2-0 overestimate it by 0.011–0.014 Å. For the highly multireference systems (B_2_, C_2_, and Cr_2_), CAS-tPBE and CAS-ftPBE perform better than CASPT2 and CASPT2-0 for B_2_ and C_2_, while CAS-ftPBE gives a similar error as CASPT2 for Cr_2_.

When comparing SP and CASSCF, we see that restricting the active space with the SP approximation only marginally alters the MAE and MAE-11 of the calculated bond distances of these diatomic molecules. The largest difference between the results obtained with CASSCF and SP was found for B_2_ and Cr_2_. In all other cases, the difference between these methods is in the range 0.002–0.007 Å, as shown in Table S1.† More importantly, there is no noticeable difference in the MAE obtained for SP-PDFT (SP-tPBE and SP-ftPBE) and MC-PDFT (CAS-tPBE and CAS-ftPBE), as shown in Fig. 3. Indeed, SP-PDFT performs equally as well as CAS-PDFT in all the cases that were tested; details are in Table S1.†

### Dissociation energies of diatomic molecules

The calculated bond dissociation energies of these twelve diatomic molecules are presented in Table 1. The dissociation energies are calculated as the difference between the potential energy of the molecule at 12 Å and the energy of the molecule at equilibrium. With CASSCF, the dissociation energies of these diatomic molecules are generally underestimated, and the MAE with respect to experimental values is 19.3 kcal mol^−1^. Without the results obtained for Cr_2_, for which it underestimates the experimental dissociation energy by 30.8 kcal mol^−1^, CASSCF has an MAE (labeled as MAE-11) of 18.2 kcal mol^−1^. This underestimation of the dissociation energy is associated with an underestimation of the force constant and is related to the excessive antibonding character of CASSCF solutions.^13^ Imposition of restrictions on the CI expansion by enforcing the SP approximation raises the MAE to 21.7 kcal mol^−1^ and the MAE to 20.8 kcal mol^−1^, corresponding to differences of 3.4 and 2.6 kcal mol^−1^, respectively, or about 11–13%. Of the methods that were tested, SP has the largest error. This is not surprising since the SP calculations use smaller active spaces than the CASSCF calculations. As discussed above, we use full valence active spaces in the CASSCF calculations, whereas the SP calculations contain only selected pairs of orbitals with no interpair excitations.

**Table 1:** The experimental dissociation energies (kcal mol^−1^) of eleven main-group diatomic molecules and the chromium dimer, Cr_2_, are compared with the calculated results obtained with several levels of theory. The MAE obtained without the results for Cr_2_ is labeled as MAE-11. For each theoretical method, the deviation of the calculated results from experimental values is given. A negative sign denotes underestimation of the bond energy, while a positive sign indicates overestimation. All experimental data are taken from ref. 40 and 42

Expt.	LiH	HF	B_2_	C_2_	CO	S_2_	SO	NH	N_2_	O_2_	F_2_	Cr_2_	MAE^a^	MAE-11
	57.7	141.3	70.0	146.0	256.2	100.8	123.5	78.5	228.5	120.3	38.2	33.9		
CAS CSFs^b^	3	15	1512	1764	1176	378	378	45	1176	378	36	28 784		
CASSCF	−13.3	−26.8	−11.0	−1.3	−4.4	−26.3	+36.5	−13.7	−16.0	−31.9	−19.1	−30.8	19.3	18.2
CASPT2	−3.9	−1.5	−3.7	+0.1	−4.0	−1.1	+1.5	+1.4	−7.1	−0.1	−0.5	−5.8	2.6	2.3
CASPT2-0	−3.5	−2.2	−4.0	−3.6	−9.6	−4.4	+3.2	−0.3	−16.5	−1.0	−1.8	−7.8	4.8	4.6
CAS-tPBE	−3.4	+4.8	−2.5	−1.3	+0.4	+6.6	+4.8	+4.5	−1.6	+8.6	+12.6	−18.5	5.8	4.6
CAS-ftPBE	−2.7	+5.7	−2.3	+3.8	−0.5	+1.9	0.3	+9.4	+4.0	+1.0	+10.8	−5.1	4.0	3.9
SP CSFs^b^	3	3	100	150	37	20	20	4	37	20	3	1516		
SP	−13.3	−26.8	−15.9	−7.1	−8.1	−26.8	+37.1	−15.2	−24.7	−32.6	−21.6	−31.2	21.7	20.8
SP-tPBE	−3.4	+4.8	+0.7	−1.2	+0.2	+6.3	+4.4	+5.0	−3.2	+8.1	+10.2	−19.4	5.6	4.3
SP-ftPBE	−2.7	+5.7	−1.2	+3.6	+0.5	+1.7	+0.6	+9.5	+2.5	+0.6	+9.0	−6.1	3.6	3.4
PBE	−4.2	+0.7	+7.0	+10.2	+11.9	+12.0	+14.6	+18.4	+14.9	+22.9	+14.8	+10.5	11.8	12.0

a MAE = mean absolute error and MAE-11 = mean absolute error without the data for Cr_2_.

b These rows give the numbers of CSFs in the CASSCF and SP calculations.

KS-PBE calculations perform much better than either CASSCF or SP in nearly all cases, as expected since neither CASSCF nor SP include dynamic correlation. However KS-PBE also has a rather large MAE (11.8 kcal mol^−1^) as well as a large MAE-11 (12.0 kcal mol^−1^). The only system for which KS-PBE approaches chemical accuracy is hydrogen fluoride (HF), a system in which the dominant configuration has a weight of 99.9%. This is by all measures a single-reference system.

The importance of including dynamic correlation for correctly computing the dissociation energies of these diatomic molecules is seen by comparing CASSCF with CAS-tPBE, CAS-ftPBE, CASPT2, and CASPT2-0 as well as by comparing SP with SP-tPBE and SP-ftPBE. The CAS-PDFT and SP-PDFT methods both perform very well for B_2_ and C_2_, which are two systems with strong multireference character (the dominant configuration has a weight of less than 80%).^41^ They reduce the MAEs and MAE-11s of CASSCF and SP by factors of about 4 and 6 respectively. For the systems presented in Table 1, the MAEs and MAE-11s of CAS-PDFT and SP-PDFT are close to those of CASPT2. When comparing CAS-PDFT and SP-PDFT to CASPT2, one has to bear in mind that the latter incorporates an empirical IPEA shift, specifically designed to improve agreement with experimental results; 2.2 (2.3) kcal mol^−1^ separates the MAE (MAE-11) of CASPT2 and CASPT2-0, indicating the importance of the empirical IPEA shift.^26^

The CAS-PDFT and SP-PDFT results are almost as good as CASPT2 and CASPT2-0. Examination of Table 1 shows that the worst results for CAS-PDFT and SP-PDFT are obtained for F_2_ (and Cr_2_ in the case of CAS-tPBE and SP-tPBE). It is particularly encouraging that SP-tPBE and SP-ftPBE essentially match CAS-tPBE and CAS-ftPBE, which are based on full-valence CASSCF solutions; this is one of the key findings of this paper, and it is important because SP-PDFT can treat much larger systems that CAS-PDFT. In principle, as CASSCF is affordable to upwards of 35 million CSFs, it should be possible to create SP solutions that approach that limit as well. As such one can envisage using SP and SP-PDFT for systems that are unaffordable for CASSCF and CAS-PDFT. As examples, SP and SP-PDFT can be used to describe the full π/π* manifold of chrysene (C_18_H_12_) as well as the full valence space of benzene–tetracyanoethylene complexes.

Two other interesting points are (1) that the results are stable as far as replacing tPBE by ftPBE or *vice versa* and (2) that ftPBE results in significant improvements in the results obtained for Cr_2_ (both for bond distances, Table S1† and for bond dissociation energies, Table 1), suggesting that it might be particularly well suited for transition metal systems.

### Potential energy curves of diatomic molecules

The ground-state potential energy curves of these twelve diatomic molecules were also scanned from near equilibrium to dissociation. Static correlation effects are generally more dominant at dissociation, and it is therefore important to test the ability of SP-PDFT to predict potential curves all the way out to this limit. The calculated potential energy curves as functions of the bond distances are presented for N_2_ and O_2_ in ESI (Fig. SI1†). CASSCF, SP, CASPT2, CAS-PDFT, and SP-PDFT all give smooth curves. The potential curves obtained with SP are similar to those obtained with CASSCF, the energies obtained with SP-tPBE are similar to those obtained with CAS-tPBE, and those obtained with SP-ftPBE are similar to those obtained with CAS-ftPBE. Thus we find that the restrictions in going from CAS to SP do not degrade the potential energy curves.

For O_2_ and N_2_ in the bonding regions (∼0.9–1.2 Å), the total electronic energy obtained with SP deviates from the CASSCF energy by about 3–10 kcal mol^−1^ as shown in Table 2. This is because some of the CSFs deleted in going from the complete active space to the separated-pair active space contribute nonnegligible amounts of dynamic correlation in these cases. At greater internuclear separations (2.5 and 5.0 Å), the differences between the total energies obtained with CASSCF and SP become small, as also shown in Table 2. In contrast, the total energies obtained with SP-tPBE and SP-ftPBE are much closer to those of CAS-tPBE and CAS-ftPBE respectively. In Table 2, we see that the largest difference between the total energies obtained with the SP-PDFT and CAS-PDFT approaches are about 1.7 kcal mol^−1^, which shows that the PDFT approach recovers the static and dynamic correlation energy that were neglected by using the approximate SP approximation in a variational wave function calculation. This is extremely encouraging. The ability of PDFT to recover these electron correlation effects is the reason why the potential energy curves obtained with SP-PDFT are closer to those obtained with CAS-PDFT in Fig. SI1† than SP is to CASSCF.

**Table 2:** Effect of imposing restrictions on the active space with the GAS scheme on the total electronic energies of N_2_, O_2_ and Cr_2_ as functions of inter-nuclear distance *R* (Å). The differences in the total electronic energies obtained with CASSCF and SP, CAS-tPBE and SP-tPBE as well as CAS-ftPBE and SP-ftPBE are reported in kcal mol^−1^, where A : B denotes the absolute value of the energy difference between A and B

	*R* (Å)	% weight of dominant configuration CASSCF : SP	CASSCF : SP	CAS-tPBE : SP-tPBE	CAS-ftPBE : SP-ftPBE
N_2_	0.9	96.0 : 96.6	7.1	1.4	1.2
	1.2	90.6 : 91.2	9.4	1.6	1.7
	2.5	12.4 : 12.1	0.54	0.26	0.27
	5.0	6.3 : 8.3	0.002	0.001	0.001
O_2_	1.1	96.0 : 96.3	3.6	0.28	0.29
	1.2	94.2 : 94.6	4.0	0.033	0.046
	2.5	34.2 : 33.8	0.26	0.21	0.20
	5.0	25.0 : 33.3	0.044	0.030	0.038
Cr_2_	1.6	57.6 : 56.4	8.8	1.0	1.1
	1.8	28.8 : 25.9	3.7	1.5	1.8
	2.6	<2 : <2	0.3	0.03	0.004
	5.0	<2 : <2	<0.001	0.002	0.002

We emphasize that the SP-PDFT and CAS-PDFT agree well both in the bonding regions of N_2_ and O_2_, where there are dominant configurations with greater than 90% weight, and in the limit of dissociation, where there are many configurations with appreciable weights. We see similar effects in Cr_2_. These results are shown in Table 2. It is also interesting to probe the origins of the agreement between SP-PDFT and CAS-PDFT by examining the approximate occupation numbers of the correlated orbitals in the SP and CASSCF solutions on which they are based, respectively. If we examine C_2_ at equilibrium, the CASSCF solution has occupation numbers of (1.9840 : 0.0136); (1.5960 : 0.3997); (1.8913 : 0.1121); (1.8911 : 0.1123), in order, for the pairs shown in Fig. 1. The SP solution has very similar occupation numbers of (1.9892 : 0.0108); (1.6051 : 0.3949); (1.8924 : 0.1074); (1.8924 : 0.1074). This suggests that the CASSCF and SP solutions result in comparable density and on-top pair density, a fact that is sufficient for quantitatively accurate SP-PDFT calculations, even though the parent SP solution is higher in energy than the analogous CASSCF solution by 0.0116 Hartrees (11.6 mH).

### Triatomic molecules

In this section, the calculated bond lengths, bond angles, and atomization energies of the two lowest energy states of methylene (CH_2_) are presented, along with the calculated adiabatic ^3^B_1_–^1^A_1_ gaps. The calculated geometry and atomization energy of ground-electronic-state ozone (O_3_) are also presented.

For CH_2_, a full valence CASSCF(6,6) wave function is used for subsequent CASPT2, CASPT2-0, and CAS-PDFT calculations. In *C*_1_ symmetry, this active space choice results in 189 and 175 CSFs for the ^3^B_1_ and ^1^A_1_ states, respectively. For SP and SP-PDFT calculations, we used the SP-4 active space, as described above. This leads to a total of 25 and 17 CSFs for the ^3^B_1_ and ^1^A_1_ states, respectively.

**Fig. 4:**
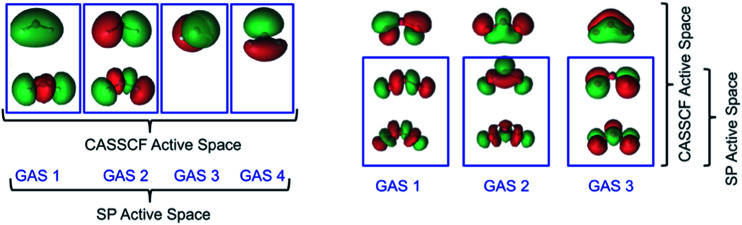
Illustrative descriptions of the CAS and SP active spaces used in CASSCF and SP calculations on ^3^B_1_ CH_2_ (*left*) and O_3_ (*right*). Refer to the text for the full descriptions of the active spaces used in the CASSCF and SP calculations.

For ozone, we used a CAS(12,9) reference for CASSCF and an SP-3 reference for the SP approximation, with the latter resulting in the reduction of the number of CSFs from 2520 to 37. In essence 98.5% of the CSFs in the CASSCF(12,9) solution are completely neglected in the SP-3 approximate wave function. These active space schemes are illustrated in Fig. 4.

#### Methylene

The structural parameters that we obtained with CASSCF are in good agreement with the CASSCF results of Apeloig *et al.*^43^ The calculated C–H bond lengths and bond angles of the ^3^B_1_ and ^1^A_1_ states of CH_2_ are compared with experimental data in Table 3. The experimental values of the C–H bond length and bond angle of the ^3^B_1_ state of CH_2_ are 1.085 Å and 135.5° respectively. For the ^1^A_1_ state, the C–H bonds are longer (1.107 Å) and the bond angle is significantly smaller (102.4°).^44^ Similarly to what was seen for the diatomic molecules, CASSCF and SP overestimate the C–H bond lengths in the ^3^B_1_ and ^1^A_1_ states of CH_2_. The calculated bond angles are also too large, as seen in Table 3.

**Table 3:** Comparison of the calculated equilibrium bond distances (*R*_e_), bond angles ( <svg xmlns="http://www.w3.org/2000/svg" version="1.0" width="10.400000pt" height="16.000000pt" viewBox="0 0 10.400000 16.000000" preserveAspectRatio="xMidYMid meet"><metadata> Created by potrace 1.16, written by Peter Selinger 2001-2019 </metadata><g transform="translate(1.000000,15.000000) scale(0.011667,-0.011667)" fill="currentColor" stroke="none"><path d="M480 1160 l0 -40 -40 0 -40 0 0 -40 0 -40 -40 0 -40 0 0 -40 0 -40 -40 0 -40 0 0 -40 0 -40 -40 0 -40 0 0 -40 0 -40 -40 0 -40 0 0 -80 0 -80 40 0 40 0 0 40 0 40 40 0 40 0 0 40 0 40 40 0 40 0 0 40 0 40 40 0 40 0 0 40 0 40 40 0 40 0 0 40 0 40 40 0 40 0 0 40 0 40 40 0 40 0 0 40 0 40 -80 0 -80 0 0 -40z M80 480 l0 -80 40 0 40 0 0 -40 0 -40 40 0 40 0 0 -40 0 -40 40 0 40 0 0 -40 0 -40 40 0 40 0 0 -40 0 -40 40 0 40 0 0 -40 0 -40 80 0 80 0 0 40 0 40 -40 0 -40 0 0 40 0 40 -40 0 -40 0 0 40 0 40 -40 0 -40 0 0 40 0 40 -40 0 -40 0 0 40 0 40 -40 0 -40 0 0 40 0 40 -40 0 -40 0 0 40 0 40 -40 0 -40 0 0 -80z"/></g></svg> ), and atomization energies (AE) of CH_2_ and O_3_ obtained at different levels of theory with experimental data. For each theoretical method, the deviation of the calculated results from experimental values is given. A negative sign denotes underestimation and a positive sign indicates overestimation of the experimental data. The calculated adiabatic singlet–triplet (S–T) gaps of CH_2_ are also presented

	CASSCF	CASPT2	CASPT2-0	CAS-tPBE	CAS-ftPBE	SP	SP-tPBE	SP-ftPBE	KS-PBE	Expt.^44,48^^,^^a^
**Methylene**										
*R*_e_ (Å)										
^3^B_1_	+0.005	−0.006	−0.005	−0.002	−0.003	+0.005	−0.003	−0.004	+0.000	1.085
^1^A_1_	+0.017	+0.005	+0.005	+0.011	+0.010	+0.023	+0.012	+0.006	+0.015	1.107

** <svg xmlns="http://www.w3.org/2000/svg" version="1.0" width="10.400000pt" height="16.000000pt" viewBox="0 0 10.400000 16.000000" preserveAspectRatio="xMidYMid meet"><metadata> Created by potrace 1.16, written by Peter Selinger 2001-2019 </metadata><g transform="translate(1.000000,15.000000) scale(0.011667,-0.011667)" fill="currentColor" stroke="none"><path d="M480 1160 l0 -40 -40 0 -40 0 0 -40 0 -40 -40 0 -40 0 0 -40 0 -40 -40 0 -40 0 0 -40 0 -40 -40 0 -40 0 0 -40 0 -40 -40 0 -40 0 0 -80 0 -80 40 0 40 0 0 40 0 40 40 0 40 0 0 40 0 40 40 0 40 0 0 40 0 40 40 0 40 0 0 40 0 40 40 0 40 0 0 40 0 40 40 0 40 0 0 40 0 40 40 0 40 0 0 40 0 40 -80 0 -80 0 0 -40z M80 480 l0 -80 40 0 40 0 0 -40 0 -40 40 0 40 0 0 -40 0 -40 40 0 40 0 0 -40 0 -40 40 0 40 0 0 -40 0 -40 40 0 40 0 0 -40 0 -40 80 0 80 0 0 40 0 40 -40 0 -40 0 0 40 0 40 -40 0 -40 0 0 40 0 40 -40 0 -40 0 0 40 0 40 -40 0 -40 0 0 40 0 40 -40 0 -40 0 0 40 0 40 -40 0 -40 0 0 40 0 40 -40 0 -40 0 0 -80z"/></g></svg> HCH (degrees)**										
^3^B_1_	−4.2	−1.9	−1.3	+0.7	+1.2	−4.1	+2.0	+3.4	+0.0	135.5
^1^A_1_	−1.1	−0.5	−0.7	+0.1	+1.0	−1.5	+0.5	+2.6	−1.6	102.4
AE of ^3^B_1_ (kcal mol^−1^)	−19.5	+0.9	−0.8	+2.5	+8.1	−22.4	+2.4	+7.1	+8.2	186.2
S–T gap (kcal mol^−1^)	+1.5	+2.7	+4.8	−1.2	−2.3	+4.5	−0.8	−2.7	+6.9	8.6

**Ozone**										
*R*_e_ (Å)	+0.005	+0.007	+0.007	−0.006	−0.005	+0.002	−0.009	−0.012	−0.003	1.278
OOO (degrees)	−0.6	−0.2	−0.4	+0.6	+0.4	−2.2	+1.8	+1.6	+1.4	116.8
AE (kcal mol^−1^)	−46.2	+2.2	−4.8	+27.7	+21.4	−81.0	+29.9	+27.5	+41.9	142.5

a To obtain these values, the thermal correction to the enthalpy at 298 K obtained by KS-PBE/aug-cc-pVTZ is added to the empirical atomization Δ*H*_298_ of CH_2_. The frequency component of this correction was scaled using a scaling factor obtained from ref. 35 for this model chemistry.

CAS-PDFT reduces the errors in the calculated structural properties of CH_2_ to within the margins provided by CASPT2 and CASPT2-0. In general, the C–H bond lengths obtained with CAS-PDFT are within 0.004–0.007 Å of the values obtained with CASPT2 and CASPT2-0. This is the case for the ^3^B_1_ and ^1^A_1_ states.

For the ^3^B_1_ state, CAS-PDFT overestimates bond angles by about 0.7–1.5° while CASPT2 underestimates them by about 1.9°. Compared with CAS-PDFT, SP-PDFT gives almost the same C–H bond lengths, and the bond angles are about 2° larger. We note that Jensen and Bunker obtained a bond angle of 133.9° for the ^3^B_1_ state.^45^ This is 1.6° below the experimental value shown in Table 3, and indicates that the results obtained with CASPT2, CASPT2-0, CAS-PDFT and SP-PDFT are within the range of available experimental data.

For the ^1^A_1_ state, CAS-PDFT overestimates the bond angle by up to 1.0°, while CASPT2 and CASPT2-0 underestimate by 0.5 and 0.7°, respectively. Similar to the situation for the ^3^B_1_ state, SP-PDFT results in slightly larger bond angles.

An earlier approach for combining MCSCF-type methods with DFT has been described by Cremer and coworkers.^46^ This method, which they call CAS-DFT, does not perform as well as CAS-PDFT and SP-PDFT for predicting the structural properties of CH_2_.^47^ It overestimates the C–H bond length of the ^3^B_1_ state by 0.017 Å. For the ^1^A_1_ state of CH_2_, it overestimates the C–H bond length by 0.031 Å.

To calculate the atomization energy of CH_2_ and O_3_, the C–H and O–O bond lengths are stretched to 12 Å, while keeping the equilibrium bond angle fixed at the value obtained with each method. (Our general conclusions remain unchanged if we use the experimental value of the bond angle.) CASSCF and SP underestimate the atomization energy of the ^3^B_1_ state of CH_2_ by about 20 kcal mol^−1^ while PBE overestimates by about 8.5 kcal mol^−1^, as shown in Table 3. Calculations with CAS-tPBE and SP-tPBE bring the error down to below 3.0 kcal mol^−1^, which is similar to CASPT2, which overestimates the bond energy by 1.2 kcal mol^−1^. Inclusion of the gradient of the on-top pair density, results in errors of 8.4 and 7.4 kcal mol^−1^ for CAS-ftPBE and SP-ftPBE, respectively, still better than KS-PBE but much worse than tPBE.

Table 3 shows that the CAS-tPBE and SP-tPBE calculations underestimate the adiabatic singlet–triplet gap by about 1.0 kcal mol^−1^ while CAS-ftPBE and SP-ftPBE calculations underestimate the separation by 2.3 and 2.7 kcal mol^−1^, respectively. The earlier CAS-DFT approach also underestimates the gap (by 1.7 kcal mol^−1^).^47^ These results are quite encouraging when compared to CASPT2 and CASPT2-0, which overestimate the separation by 2.7 and 4.8 kcal mol^−1^, respectively.

#### Ozone

The ozone molecule has been studied with a large number of quantum-mechanical methods.^11,49–51^ We highlight the work of Vogiatzis and coworkers in which they showed that a GAS-2(12,9)-1e active space provides the same MAE as CASSCF(12,9) for the computed vertical excitation energies, ionization potential, and electron affinity of O_3_.^11^ The GAS-2(12,9)-1e notation corresponds to two subspaces containing 12 electrons in 9 orbitals with one excitation allowed between the subspaces.

**Fig. 5:**
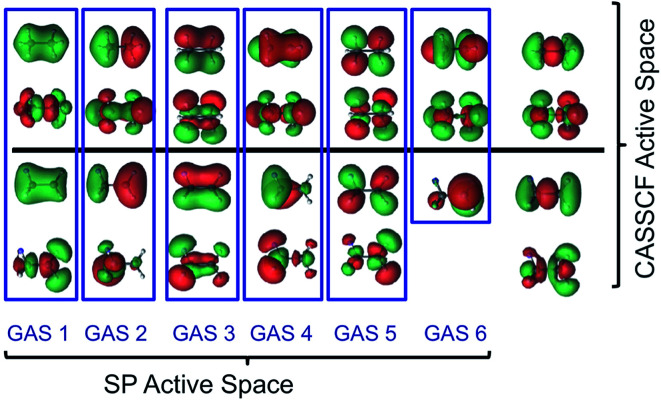
Illustrative descriptions of the CAS and SP active spaces used in CASSCF and SP calculations on: ethane (top) and the ethyl radical (bottom). Notice that the C–C orbitals are not included in SP subspaces as we are concerned only with C–H bond dissociation.

In the present work, we have used an even more restrictive framework, namely SP-3, which becomes GASSCF-3(6,6)-0e in the general notation. The first two subspaces each contain a coupled pair of σ and σ* orbitals while the third space contains a coupled pair of π and π* orbitals. In contrast, we placed 12 electrons in 9 orbitals for the CASSCF calculations. The nine orbitals are those formed by combination of the 2p_*x*_, 2p_*y*_, and 2p_*z*_ orbitals of the three oxygen atoms, as shown in Fig. 5. The dominant configuration in the CASSCF(12,9) wave function has a weight of only 84%, showing that this system has significant multi-reference character.

Table 3 shows that the optimized geometry of O_3_ obtained with CASSCF is in good agreement with the results of Tsuneda *et al.*,^51^ who used a similar active space with the cc-pVTZ basis sets augmented with s, p, and d diffuse functions. Also, the structural parameters obtained with CASPT2 are in agreement with the reports of Ljubic and Sabljic, who used the same active space.^50^ The bond lengths and bond angle obtained with SP-PDFT are similar to those obtained with CAS-PDFT, despite the fact that the underlying SP wave function contains only about 1.4% of the number of CSFs in the CASSCF solution. CAS-PDFT slightly underestimates the O–O distances and slightly overestimates the bond angle, while CASPT2 and CASPT2-0 have opposite behaviors.

Krishna and Jordan have previously reported that CASSCF underestimates the atomization energy of O_3_ by about 57.7 kcal mol^−1^.^52^Table 3 shows that CASSCF and the SP approximation are both poor for calculating the atomization energy of O_3_. These are the two methods that do not attempt to include most of the dynamic electron correlation. On the other hand, PBE overestimates the atomization energy by about 42 kcal mol^−1^ but CAS-tPBE and CAS-ftPBE reduce the error of PBE by 14 and 20 kcal mol^−1^, respectively. SP-tPBE and SP-ftPBE behave similarly to CAS-tPBE and CAS-ftPBE respectively. However, CASPT2 and CASPT2-0 perform best for predicting the atomization energy of O_3_.

### C–H bond dissociation energies in organic compounds

In this section we study C–H bond dissociation in acetylene, ethylene, and ethane, that is:C_2_H_2_ → C_2_H˙ + H˙C_2_H_4_ → C_2_H_3_˙ + H˙C_2_H_6_ → C_2_H_5_˙ + H˙

The calculated energies for these reactions are compiled in Table 4, where they are compared to experimental values estimated by adding the thermal correction to the enthalpy at 298 K obtained by KS-PBE/aug-cc-pVTZ to the empirical Δ*H*_298_ reported by Blanskby and Ellison.^53^ The frequency component of this correction was scaled using scaling factors obtained from ref. 35 for this model chemistry. We used full valence active spaces for the CASSCF calculations in this table: CAS(10,10), CAS(9,9), CAS(12,12), CAS(11,11), CAS(14,14) and CAS(13,13) for acetylene, ethynyl, ethylene, vinyl, ethane and ethyl respectively. With *C*_1_ symmetry, these active spaces result in 19 404, 8820, 226 512, 104 544, 2 760 615 and 1 288 287 CSFs respectively. In Table 4 we report the number of CSFs only for the parent compound and not for the dissociation radical species. The active spaces used in SP and SP-PDFT calculations are presented in the computational details section and are illustrated for ethane and the ethyl radical in Fig. 5. Only orbitals with significant C–H character are included in the SP active spaces. As previously noted, we used SP-3, SP-3, SP-4, SP-4, SP-6 and SP-6 active spaces for the acetylene, ethynyl, ethylene, vinyl, ethane and ethyl, respectively. These result in 37, 17, 150, 76, 3012 and 1704 CSFs respectively, a significant reduction compared with the full CASSCF calculation.

**Table 4:** The calculated C–H dissociation energies (kcal mol^−1^) of three organic compounds obtained with different levels of theory are compared with experimental data. For each theoretical method, the deviations of the calculated results from experimental values are given. A negative sign denotes underestimation and a positive sign indicates overestimation of the experimental data

	CASSCF	CAS CSFs^a^	CASPT2	CASPT2-0	CAS-tPBE	CAS-ftPBE	SP	SP CSFs^a^	SP-tPBE	SP-ftPBE	KS-PBE	Expt.^b^
Acetylene	123.7	19 404	136.1	134.8	140.7	141.4	115.7	37	141.5	141.3	138.2	140.9
Error	−17.2		−4.8	−6.1	−0.2	+0.5	−25.2		+0.6	+0.4	−2.7	
Ethylene	106.3	226 512	114.9	113.7	116.7	116.9	93.8	150	117.4	116.0	113.6	119.5
Error	−13.2		−4.6	−5.8	−2.8	−2.6	−25.7		−2.1	−3.5	−5.9	
Ethane	99.7	2 760 615	106.4	105.1	106.1	105.7	68.8	3012	112.3	111.3	104.6	110.4
Error	−10.7		−4.0	−5.3	−4.3	−4.7	−41.6		+1.9	+0.9	−5.8	
MSE	−13.7		−4.5	−5.7	−2.4	−2.3	−30.8		0.1	−0.7	−4.8	
MAE	13.7		4.5	5.7	2.4	2.6	30.8		1.5	1.6	4.8	

a These columns give the numbers of CSFs in the CASSCF and SP calculations for the compound.

b Thermal correction to the enthalpy at 298 K obtained by KS-PBE/aug-cc-pVTZ are added to empirical enthalpy Δ*H*_298_ data.^53^

In all the three cases presented in Table 4, CAS-PDFT performs much better than CASPT2, CASPT2-0, or PBE, and SP-tPBE and SP-ftPBE do even better with MAEs of only 1.6 and 1.7 kcal mol^−1^, respectively. This is another demonstration that PDFT effectively recovers correlation that is left behind by the active space restrictions of the SP approximation, even though we were very aggressive in including only a small number of pairs. Fig. S2† shows that the SP-PDFT potential energy curves for C–H cleavage in acetylene, ethylene, and ethane match those obtained with CASPT2 and CAS-PDFT very well, so the active space restrictions do not distort the shape of the potential energy curves.

In general we are presenting the SP-PDFT results for just one small active space, as previously indicated. We did do some testing to see the effect of using different choices of which pair of orbitals to include, and we found that the effect of adding or removing spectator pairs was small. For example we used SP-5 for ethylene and vinyl and found a difference in the calculated C–H dissociation energy of only 0.2 kcal mol^−1^ as compared to the SP-4 results presented in the table.

### Barrier heights for pericyclic reactions

**Fig. 6:**
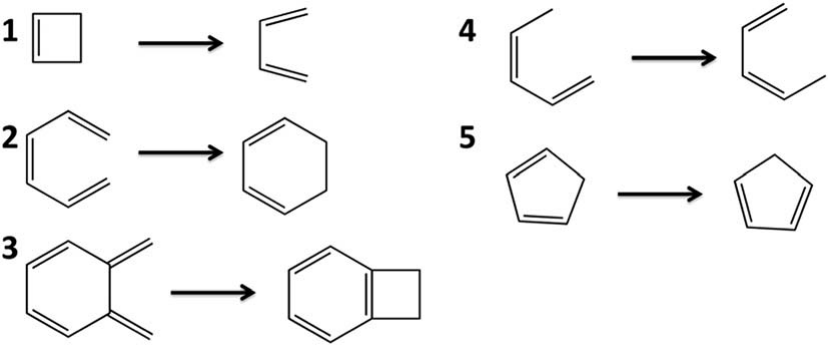
The barriers for these five pericyclic reactions were calculated with SP-PDFT and other theoretical methods.

We have previously shown that CAS-tPBE reduces the average error of PBE by a factor of 2.7 for predicting the forward and reverse barrier heights for chemical reactions involving small molecules.^19^ CASPT2 however was found to have a lower MAE than CAS-tPBE. Houk and coworkers have collected datasets of the barrier heights of pericyclic reactions.^33,54^ These datasets can be used to benchmark computational approaches. In Table 5, we compare the calculated barriers for five pericyclic reactions with experimental data taken from the dataset of Houk and coworkers.^33,54^ These reactions are shown in Fig. 6.

**Table 5:** The calculated barriers (kcal mol^−1^) of five pericyclic reactions computed with different theoretical methods are compared with experimental data. For each theoretical method, the deviations of the calculated results from experimental values are also given. A negative sign denotes underestimation and a positive sign indicates overestimation of the experimental data

	CASSCF	CAS CSFs^a^	CASPT2	CASPT2-0	CAS-tPBE	CAS-ftPBE	SP	SP CSFs^a^	SP-tPBE	SP-ftPBE	KS-PBE	Expt.^b^
**1**	36.0	20	34.0	32.5	36.0	36.2	36.6	10	36.2	36.3	32.1	33.6
Error	+2.4		+0.4	−1.1	+2.4	+2.6	+3.0		+2.6	+2.7	−1.5	
**2**	45.3	175	28.8	27.7	30.0	30.0	58.3	37	30.3	30.8	25.5	30.2
Error	+15.1		−1.4	−2.5	−0.2	−0.2	+28.1		+0.1	+0.6	−4.7	
**3**	39.1	1764	25.5	24.4	25.8	25.8	52.1	150	25.5	25.9	23.2	29.5
Error	+9.6		−4.0	−5.2	−3.7	−3.7	22.0		−4.0	−3.6	−6.3	
**4**	50.8	20	36.4	35.4	31.9	33.1	60.1	10	32.0	32.9	31.3	38.8
Error	+12.0		−2.4	−3.4	−6.9	−5.7	+21.3		−6.8	−5.9	−7.5	
**5**	43.9	20	25.5	25.4	21.0	22.3	40.7	10	20.9	21.9	22.8	25.8
Error	+18.1		−0.3	−0.4	−4.8	−3.5	+14.9		−4.8	−3.8	−3.0	
MSE	11.4		−1.5	−2.5	−2.6	−2.1	17.9		−2.6	−2.0	−4.6	
MAE	11.4		1.7	2.5	3.6	3.1	17.9		3.7	3.2	4.6	

a These columns give the numbers of CSFs in the CASSCF and SP calculations.

b Scaled zero point energy corrections obtained at the B3LYP/6-31G(d) level are added to experimental Δ*H*^‡^_0K_ data to obtain the last column.^33,54^

SP and CASSCF overestimate the reaction barriers by 15 kcal mol^−1^ or more, in nearly all cases. Table 5 shows that the only exception is for reaction 1, for which they overestimate it by only 2.4 and 3.0 kcal mol^−1^ respectively. SP-PDFT and CAS-PDFT greatly improve on CASSCF and SP, reducing the MAEs by factors of 3–6. This is similar to the situation found for reactions involving small molecules.^19^ The MAEs of SP-PDFT and CAS-PDFT are still somewhat large, at least when compared to CASPT2. However, regarding the central topic of this manuscript, we find that the calculated reaction barriers are stable to the use of a restricted SP wave function; that is, SP-PDFT and CAS-PDFT yield similar results for each reaction and overall have similar MAEs.

### Open-shell singlet systems

**Fig. 7:**
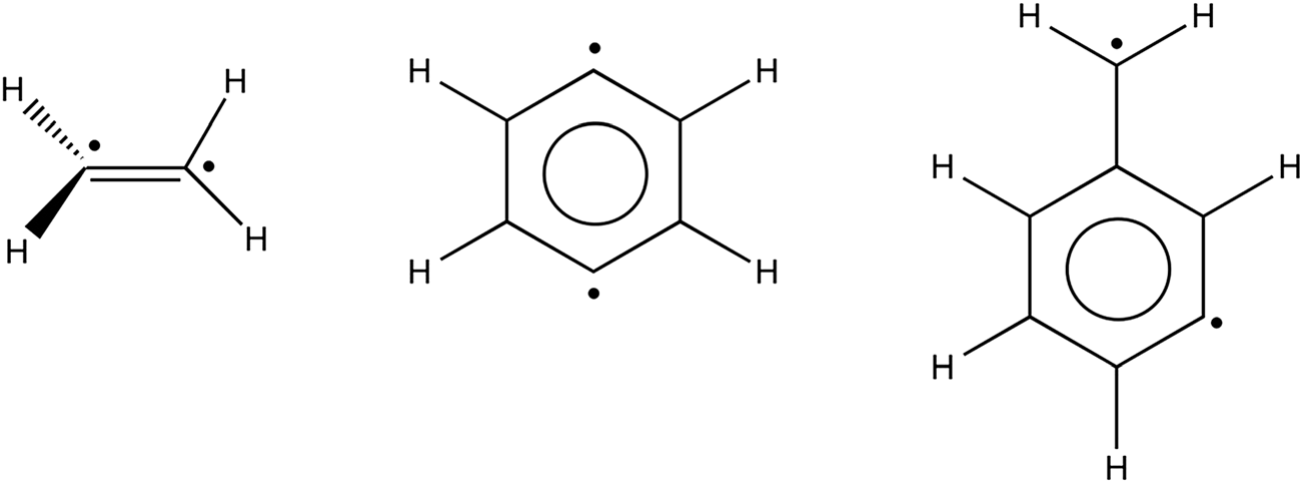
Illustrations of the structures of twisted ethylene, 1,4-didehydrobenzene and α,3-didehydrotoluene.

Cremer and coworkers have previously used UDFT, broken-symmetry UDFT, and CAS-DFT^46^ to study open-shell singlet diradicals.^47^ Specifically, they studied the energy of the ^1^B_1_ state of twisted ethylene relative to planar ethylene. In addition, they also studied the singlet–triplet gaps of 1,4-didehydrobenzene and α,3-didehydrotoluene, which are shown in Fig. 7. They found that CAS-DFT predicts the ^1^A′′ state of α,3-didehydrotoluene to be lower in energy than the ^3^A′′ state. Also the ^1^A_g_ state of 4-didehydrobenzene is predicted to be lower in energy than the ^3^B_1u_ state. These state orderings are in agreement with experimental data.^55,56^ In Table 6, we compare the results obtained when PBE, CASSCF, SP, SP-PDFT, CAS-PDFT and CASPT2 are used to carry out similar computations to those performed by Cremer and coworkers.^47^ We used CAS(8,8) and SP-4 active spaces in the calculations on the singlet and triplet states of 1,4-didehydrobenzene and α-3-dide-hydrotoluene. For the KS-DFT computations, the singlet states were treated as unrestricted broken-symmetry solutions. For planar and twisted ethylene, we used CAS(12,12) and SP-1 active spaces, respectively.

**Table 6:** The calculated energy separation (kcal mol^−1^) of twisted and planar ethylene (**A**) and the singlet–triplet gaps of 1,4-didehydrobenzene (**B**) and α,3-didehydrotoluene (**C**)

	CASSCF	CAS CSFs^a^	CASPT2	CASPT2-0	CAS-tPBE	CAS-ftPBE	SP	SP CSFs^a^	SP-tPBE	SP-ftPBE	KS-PBE^b^	Expt.
**A** c	73.1	226 512	69.7	67.6	72.6	69.5	72.0	3	74.3	72.5	65.8	
**B** c	−2.6	2352; 1764	−4.7	−4.5	−3.9	−4.0	−3.7	204; 200	−3.9	−4.0	−3.6	−3.5 ± 0.5 (ref. 55)
**C** c	−2.9	2352; 1764	−1.9	−1.7	−0.3	−0.4	+0.7	204; 200	−0.3	−0.3	−0.7	−2.5 ± 2.5 (ref. 56)

a These columns give the numbers of CSFs in the CASSCF and SP calculations. For **B** and **C**, we give the number of CSFs in the triplet state first.

b We compare the variational energies obtained at the PBE level.

c For **A**, we give the energy of twisted ethylene relative to planar ethylene. For **B** and **C**, we give the energies of the singlet states relative to the triplet state.

For the gap between twisted and planar ethylene, SP-tPBE and SP-ftPBE provide similar results that are 2–3 kcal mol^−1^ above those obtained with CAS-tPBE and CAS-ftPBE respectively, as shown in Table 6. The results obtained with CAS-ftPBE and CASPT2 are the same. The results obtain for the fully translated functionals improve upon CAS-tPBE and SP-tPBE. Lischka and coworkers obtained a value of 69.2 kcal mol^−1^ at the MR-CISD+Q/SA-3-RDP level while using similar basis sets.^34^ This is close to the values obtained by CAS-ftPBE and CASPT2.

For 1,4-didehydrobenzene, the results obtained with CAS-tPBE, CAS-ftPBE, SP-tPBE and SP-ftPBE fall within the error bar of the experiment, which is −3.5 ± 0.5 kcal mol^−1^. In contrast, CASPT2 and CASPT2-0 fall outside this range; they overestimate even as compared to the high end of the experimental results^55^ by 0.7 and 0.5 kcal mol^−1^, respectively. CAS-DFT predicted an energy separation of −2.6 kcal mol^−1^ between the ^1^A_g_ and ^3^B_1u_ states of 1,4-didehydrobenzene.^47^ For α,3-didehydrotoluene, CAS-tPBE, CAS-ftPBE, SP-tPBE and SP-ftPBE correctly predict that the ^1^A′′ state is more stable than the ^3^A′′ state. The separations provided by all these methods are similar and fall within the range of available experimental data. They are however about 1.5 kcal mol^−1^ smaller than the separations predicted by CASPT2 and CASPT2-0. Unfortunately, available experimental reports only indicate that the splitting should be lesser than 5.0 kcal mol^−1^.

## Concluding remarks

The present paper contains new methods for both wave function theory and density functional theory. Starting with wave function theory, we have presented a systematic way to choose the active space in generalized-active-space self-consistent-field (GASSCF) theory. This new way of choosing the active space is called the separated-pair (SP) approximation. The method is intermediate between generalized valence bond (GVB) theory and complete active space self-consistent-field (CASSCF) theory. The SP wave function is a truncation of CASSCF, obtained by partitioning the CAS active space into an arbitrary number of generalized-active-space (GAS) subspaces that each contain at most two orbitals, and inter-subspace excitations are excluded. In the examples, only pairs required to describe a particular bond-breaking process are included in the GAS subspaces; all other orbitals are treated as doubly occupied in all configurations. With such a choice, the SP methods can be used for large systems for which conventional CASSCF calculations are unaffordable. Just as for GVB and CASSCF, the precise choice of active space in the SP approximation is not completely unambiguous because in all three methods one must decide which orbitals to correlate. The orbitals to be correlated are an individual choice, although we expect that the most useful choice will usually be a bonding orbital and the corresponding antibonding orbital. In the present paper we include the orbitals involved in bond breaking and in some cases also additional orbitals closely close coupled to the bond breaking. A general objective might be to include the orbitals responsible for nondynamic correlation, which is also called static correlation, strong correlation, and near-degeneracy correlation. Although dynamic correlation tends to be very similar across systems, nondynamic correlation is usually system-specific. Therefore, a practical multi-configuration approach may well have to be applied in a case-by-case manner, sometimes requiring chemical insight. But the examples presented here show that simple considerations lead to reasonably accurate results for a set of diverse cases and significantly reduce the computational cost of specific problems. The definition and exploration of SP may be useful for all methods that need to start from a strongly correlated reference wave function.

We subsequently considered whether the SP approximation is useful for multi-configuration pair-density functional theory (MC-PDFT), and to put this in context we first contrast MC-PDFT to Kohn–Sham density functional theory (KS-DFT). In KS-DFT, ones represents the density by a Slater determinant, and one writes the total energy as the sum of the kinetic energy computed from the Slater determinant by standard wave function methods, the Coulomb energy computed classically from the density, and a remainder. The remainder is a functional of the density and is called the exchange–correlation energy, and it includes not just the deviation of the true potential energy from the Coulomb energy computed classically from the density, but also the deviation of the true kinetic energy from the Slater-determinant kinetic energy. In MC-PDFT, we represent the density and the on-top pair density by a multi-configurational wave function, and we write the total energy as the sum of the kinetic energy computed from the multi-configurational wave function by standard wave function methods, the Coulomb energy computed classically from the density, and a remainder. The remainder is a functional of the density and the on-top pair density and is called the on-top energy, and it includes both the deviation of the true potential energy from the Coulomb energy computed classically from the density and the deviation of the true kinetic energy from the multi-configurational-wave-function kinetic energy. In most previous attempts to combine multi-configurational wave functions with density functional theory, one writes the total energy as the sum of the energy computed by wave function theory from the multi-configurational wave function plus a remainder. Because the energy computed by wave function theory from the multi-configurational wave function includes some of the effect of electron correlation on the true potential energy, one must be careful not to include this portion of the correlation energy in the remainder; this can be called the double counting problem. Because we use only the kinetic energy of the multi-configurational wave function, we avoid this double counting problem. Note though that we do not know an exact on-top functional, just as an exact exchange–correlation functional is not known in KS-DFT, so our treatment is not exact. A major goal of both KS-DFT and MC-PDFT is to find a better approximation to the corresponding exact functional. One motivation for MC-PDFT is that it might be “easier” to find a good on-top functional than to find a good exchange–correlation functional for two reasons: (1) our kinetic energy is based on a representation that better conforms to the true wave function when (as is often the case) the system is inherently multi-configurational (as, for example, for describing the breaking of a covalent bond), and (2) our functional is allowed to depend on the on-top pair density, which brings in extra information. Many years of development have gone into modern exchange–correlation functionals,^57^ whereas for on-top functionals we are still using first-generation approximations. The present paper, however, is not about improving the functional but rather about testing how many and what kind of configurations need be present in the multi-configurational wave function in order obtain reasonable results with simple density functionals. We found that the new SP approximation, discussed in the previous paragraph for wave function theory, provides an economical multi-configurational wave function that yields good accuracy with MC-PDFT. Thus we have presented a version of MC-PDFT called separated-pair pair-density functional theory, abbreviated SP-PDFT. The SP-PDFT method uses a separated-pair (SP) wave function to generate the kinetic and classical Coulomb contributions to the total electronic energy, and the remainder of the total electronic energy is computed from a functional of the total density and the on-top pair density taken from the SP wave function. The SP-PDFT methods can therefore be used for large systems for which conventional CASSCF calculations, CASPT2, and CAS-PDFT are unaffordable.

Sometimes the SP approximation wave function calculations agree well with the CASSCF ones; in other cases they are less accurate, as would be expected. But even in cases where the energetic results obtained by wave function theory from the SP approximation are less accurate than those obtained by CASSCF, we show that the SP approximation is able to produce an accurate enough kinetic energy and on-top pair density that the SP-PDFT results are in generally good agreement with the CAS-PDFT results. The tests included in this article include structural properties and bond dissociation energies of eleven diatomic and two triatomic molecules, the C–H dissociation energies of prototypical organic systems, the reaction barriers of pericyclic reactions, and the description of open-shell singlet species. In all the cases that were tested, SP-PDFT provides approximately the same accuracy as CAS-PDFT. In most cases, both SP-PDFT and CAS-PDFT provide similar levels of accuracy as the much more expensive CASPT2 approach; the only exception to this is for the reaction barriers of pericyclic reactions.

The key result for wave function theory is that the SP approximation often agrees quite well with CASSCF, at greatly reduced cost, and this extends the usefulness of the method to bigger systems. The key result for density functional theory is that the quality of results obtained from MC-PDFT calculations remains largely unchanged even with drastic reductions in the number of included CSFs, as we have in made in the SP-PDFT variant of the method. In addition the SP-PDFT approach, just as is the more general MC-PDFT framework, is free of double-counting of electron correlation energies. This double-counting problem plagues nearly all other hybrid approaches for combining CASSCF and KS-DFT.

Future work that one can anticipate includes testing the performance of the SP and SP-PDFT methods for transition metal complexes, developing better on-top functionals of the MCSCF density and on-top pair density, and developing an orbital optimization algorithm that includes the on-top functional in the self-consistent-field step.

## Supplementary Material

SC-007-C5SC03321G-s001
