# Supplementary material for: Separated-pair approximation and separated-pair pair-density functional theory
Source: Chem Sci. 2015 Dec 16;7(3):2399–413. doi: 10.1039/c5sc03321g (PMC6003605; doi:10.1039/c5sc03321g)
Supplement: SC-007-C5SC03321G-s001 [file SC-007-C5SC03321G-s001.pdf]

Electronic Supplementary Material (ESI) for Chemical Science.
This journal is © The Royal Society of Chemistry 2015
Please do not adjust margins

Supporting Information for "The Separated-Pair Approximation and Separated-Pair Pair-Density Functional Theory"

S. O. Odoh,[a] G. L. Manni,[a,b] R. K. Carlson,[a] D. G. Truhlar[a,*] and L. G. Gagliardi[a,*]

Geometries

Pericyclic Reaction 1

Reactant
H 0.000000 -1.420641 1.601473
C 0.000000 0.670170 0.814819
C 0.000000 -0.670170 0.814819
C 0.000000 0.786311 -0.699697
C 0.000000 -0.786311 -0.699697
H 0.890109 -1.246599 -1.146101
H -0.890109 -1.246599 -1.146101
H 0.000000 1.420641 1.601473
H -0.890109 1.246599 -1.146101
H 0.890109 1.246599 -1.146101
TS
H 1.881849 -1.100924 -0.435440
C 1.064845 -0.626082 0.119166
C -1.065620 -0.625274 -0.119189
C 0.683760 0.732882 -0.083831
C -0.682927 0.733403 0.083647
H -1.882719 -1.099377 0.435915
H -1.338688 1.542124 0.403314
H 0.870699 -1.086421 1.080648
H -0.871987 -1.086153 -1.080502
H 1.340495 1.541172 -0.402694

Pericyclic Reaction 2

Reactant

C	-0.318884000	0.000066000	2.894521000
H	0.641335000	0.000179000	3.405963000
H	-1.205045000	0.000070000	3.521831000
C	-0.400823000	-0.000062000	1.553914000
H	-1.386558000	-0.000136000	1.092276000
C	0.757542000	-0.000064000	0.677687000
H	1.723262000	0.000044000	1.182403000
C	0.757542000	-0.000037000	-0.677687000
H	1.723262000	0.000084000	-1.182403000
C	-0.400823000	0.000002000	-1.553914000
H	-1.386558000	-0.000050000	-1.092276000
C	-0.318884000	0.000046000	-2.894521000
H	0.641335000	0.000081000	-3.405963000
H	-1.205045000	0.000024000	-3.521831000

TS

C	-1.203716	1.140653	-0.106083
C	0.119843	1.482489	0.188734
C	1.242319	0.702964	-0.106083
C	1.242319	-0.702964	-0.106083
C	0.119843	-1.482489	0.188734
C	-1.203716	-1.140653	-0.106083
H	0.296276	2.340488	0.841280
H	2.220000	1.175106	-0.016579
H	2.220000	-1.175106	-0.016579
H	0.296276	-2.340488	0.841280
H	-1.999204	-1.641147	0.447050
H	-1.467749	-0.936255	-1.131155
H	-1.999204	1.641147	0.447050
H	-1.467749	0.936255	-1.131155

Pericyclic Reaction 3

Reactant

H	-0.144883000	1.244000000	2.790787000
C	-0.079370000	0.721429000	1.840229000
C	-0.128693000	1.408985000	0.677482000
C	0.079370000	-0.721429000	1.840229000

Please do not adjust margins

Please do not adjust margins

C	0.128693000	-1.408985000	0.677482000
H	0.144883000	-1.244000000	2.790787000
H	-0.221218000	2.492345000	0.677508000
C	-0.006727000	0.748467000	-0.621344000
C	0.006727000	-0.748467000	-0.621344000
H	0.221218000	-2.492345000	0.677508000
C	0.128693000	1.497447000	-1.739509000
C	-0.128693000	-1.497447000	-1.739509000
H	0.089546000	2.581730000	-1.689010000
H	0.294457000	1.064480000	-2.720427000
H	-0.294457000	-1.064480000	-2.720427000
H	-0.089546000	-2.581730000	-1.689010000

TS

H	0.696138000	2.508565000	0.264710000
C	0.687715000	1.425476000	0.168400000
C	0.687709000	-1.425477000	-0.168399000
C	-0.516000000	0.709437000	-0.014178000
C	1.867142000	0.702428000	0.105773000
C	1.867138000	-0.702434000	-0.105775000
C	-0.516002000	-0.709434000	0.014188000
C	-1.855138000	1.105047000	-0.302705000
H	2.820250000	1.219913000	0.179461000
H	2.820245000	-1.219922000	-0.179473000
H	-2.323464000	-0.714596000	1.196424000
H	0.696128000	-2.508565000	-0.264714000
H	-2.323460000	0.714584000	-1.196421000
H	-2.295179000	2.031077000	0.075446000
H	-2.295185000	-2.031066000	-0.075465000
C	-1.855143000	-1.105042000	0.302701000

Pericyclic Reaction 4

Reactant

C -0.907602 0.000000 -1.959333
C 0.448390 0.000000 -1.314843
C 0.737123 0.000000 0.000000
C -0.208361 0.000000 1.108435
C 0.156236 0.000000 2.399613
H 1.288112 0.000000 -2.009782
H 1.788622 0.000000 0.288446
H -1.269802 0.000000 0.866648
H -0.576276 0.000000 3.201222
H 1.203097 0.000000 2.696162
H -1.726378 0.000000 -1.235084
H -1.031048 0.879920 -2.605421
H -1.031048 -0.879920 -2.605421

TS

C -0.906955 1.309420 0.015864
C -0.906955 -1.309420 0.015864
C 0.507865 1.211640 0.015864
C 0.507865 -1.211640 0.015864
C 1.185505 0.000000 -0.142456
H -1.206145 0.000000 0.498374
H -1.333655 2.181680 0.515504
H -1.333655 -2.181680 0.515504
H -1.451815 1.068700 -0.896696
H -1.451815 -1.068700 -0.896696
H 1.091195 2.046030 0.405514
H 1.091195 -2.046030 0.405514
H 2.270755 0.000000 -0.073006

Pericyclic Reaction 5

Reactant

H -1.880936 0.878816 0.000098
C -1.218209 0.000144 -0.000003
C -0.283259 0.000058 -1.181349
C 0.993103 -0.000300 -0.734692
C 0.993099 0.000400 0.734696
C -0.283264 -0.000375 1.181348
H -1.881417 -0.878142 -0.000107
H -0.610081 -0.000604 2.214541
H -0.610071 0.000119 -2.214544
H 1.886846 -0.000395 -1.349345

This journal is © The Royal Society of Chemistry 2015

Please do not adjust margins

Please do not adjust margins

H 1.886834 0.000643 1.349358
TS
C 0.745102 -0.040400 -0.937637
C -0.745573 -0.040390 -0.937240
C 1.149270 -0.018772 0.412626
C -1.149046 -0.018741 0.413240
C 0.000315 -0.008670 1.215464
H -0.000331 1.017963 -1.168788
H 1.353558 -0.191751 -1.821052
H -1.354605 -0.191775 -1.820266
H 2.174927 0.032335 0.756061
H -2.174548 0.032221 0.757150
H 0.000593 0.062844 2.298174

Planar Ethylene

C	-1.070792097	0.000000000	-0.108981896
C	-1.070792097	0.000000000	1.228018104
H	-1.070792097	0.927535970	-0.668057942
H	-1.070792097	-0.920230942	-0.680002048
H	-1.070792097	0.927535970	1.787094149
H	-1.070792097	-0.920230942	1.799038255

Twisted ethylene

C	-0.888566203	0.000000000	-1.031498053
C	-0.888566203	0.000000000	0.337501947
H	0.023128225	0.000000000	-1.648761586
H	-1.800260632	0.000000000	-1.648761586
H	-0.888566203	0.911694428	0.954765480
H	-0.888566203	-0.911694428	0.954765480

α,3-didehydrotoluene

Triplet

C	0.190636	-1.188329	0.000000
C	0.974710	-0.005882	0.000000
C	-1.193747	-1.141554	0.000000
H	-1.764361	-2.065844	0.000000
C	0.287802	1.242844	0.000000
C	-1.867651	0.089913	0.000000
H	0.853472	2.170665	0.000000
H	-2.951428	0.138462	0.000000
C	-1.078067	1.221271	0.000000
H	0.699337	-2.148129	0.000000
C	2.386133	-0.061761	0.000000
H	2.906164	-1.011827	0.000000
H	2.980558	0.843516	0.000000

Singlet

C	-0.169550	1.262266	-0.096111
C	-0.996960	0.107084	-0.163647
C	1.206318	1.164511	0.022693
H	1.808502	2.067153	0.071686
C	-0.356759	-1.166843	-0.104483
C	1.832588	-0.091691	0.080618
H	-0.954308	-2.073206	-0.153065
H	2.909862	-0.179405	0.173776
C	1.004292	-1.195198	0.012734
H	-0.641105	2.239846	-0.139821
C	-2.395123	0.214285	-0.284493
H	-2.878818	1.182524	-0.329039
H	-3.020689	-0.668410	-0.335878

1,4-didehydrobenzene

Triplet

C	-0.372103	0.366454	-1.449859
C	-1.476309	0.027675	-0.697734
C	0.789674	0.722895	-0.748247
H	1.699213	1.001948	-1.272489
C	-1.546916	0.006012	0.678594
C	0.719067	0.701233	0.628081
H	-2.456454	-0.273041	1.202835
C	-0.385139	0.362454	1.380205
H	-0.364408	0.368814	2.466248
H	-0.392834	0.360093	-2.535902

Singlet

C	-0.379290	0.364248	-1.444216

This journal is © The Royal Society of Chemistry 2015

Please do not adjust margins

Please do not adjust margins

C	-1.486871	0.024435	-0.704113
C	0.788596	0.722564	-0.738916
H	1.688804	0.998755	-1.279203
C	-1.545838	0.006343	0.669262
C	0.729629	0.704473	0.634459
H	-2.446045	-0.269847	1.209549
C	-0.377952	0.364659	1.374563
H	-0.375170	0.365512	2.460176
H	-0.382071	0.363395	-2.529830

This journal is © The Royal Society of Chemistry 2015

Please do not adjust margins

Please do not adjust margins

Table S1: Comparison of the calculated and experimental equilibrium bond distances of several diatomic molecules obtained at various levels of theory. The experimental distances are given in Angstroms while the deviation of the calculated results are given. A + sign denotes over-estimation while a – sign denotes underestimation.

	LiH	HF	B_2	C_2	CO	S_2	SO	NH	N_2	O_2	F_2	Cr_2	MAE
Expt.	1.596	0.917	1.590	1.243	1.128	1.889	1.481	1.036	1.098	1.208	1.412	1.680	
CAS CSFs	3	15	1512	1764		378	378	45	1176	378	36	28784	
CASSCF	+0.039	+0.000	+0.026	+0.012	+0.007	+0.041	+0.036	+0.011	+0.007	+0.008	+0.048	+1.520	0.023
CASPT2	+0.019	+0.003	+0.013	+0.008	+0.008	+0.024	+0.015	+0.003	+0.007	+0.004	+0.011	+0.060	0.011
CASPT2-0	+0.019	+0.003	+0.015	+0.009	+0.009	+0.028	+0.018	+0.006	+0.008	+0.007	+0.014	+0.720	0.013
CAS-tPBE	+0.009	+0.009	+0.006	+0.005	+0.009	+0.017	+0.013	+0.013	+0.005	-0.001	-0.023	+0.020	0.010
CAS-ftPBE	+0.015	+0.009	+0.001	+0.001	+0.009	+0.014	+0.006	+0.008	+0.003	-0.004	-0.021	+0.020	0.008
SP CSFs	3	3	100	150		20	20	4	37	20	3	1516	
SP	+0.039	+0.000	-0.001	0.005	+0.004	+0.041	+0.036	+0.011	+0.005	+0.008	+0.055	+1.520	0.020
SP-tPBE	+0.009	+0.009	+0.001	+0.005	+0.006	+0.017	+0.012	=0.011	+0.005	+0.002	-0.025	+0.030	0.010
SP-ftPBE	+0.015	+0.009	-0.002	+0.001	+0.006	+0.013	+0.005	+0.009	+0.003	-0.005	-0.023	-0.010	0.009
PBE	+0.083	+0.015	+0.028	+0.012	+0.009	+0.032	+0.034	+0.014	+0.005	+0.012	+0.001	+0.023	0.024

This journal is © The Royal Society of Chemistry 2015

Please do not adjust margins

Please do not adjust margins

Description of the active spaces used in CASSCF and SP calculations on all the compounds and reactions tested in this work.

Molecule	CAS Active Space	SP active space
LiH	2,2	SP-1
HF	8,5	SP-1
B_2	6,8	SP-4 (two GAS spaces containing one orbital each and one electron each)
C_2	8,8	SP-4
CO	10,8	SP-3
S_2	12,8	SP-3
SO	12,8	SP-3
NH	6,5	SP-3 (two GAS spaces containing one orbital each and one electron each)
N_2	10,8	SP-3
O_2	12,8	SP-3
F_2	14,8	SP-1
Cr_2	12,12	SP-6
CH_2	6,6	SP-4 (two GAS spaces containing one orbital each and one electron each)
O_3	12,9	SP-3
Acetylene	10,10	SP-3
Ethynyl radical	9,9	SP-3
Acetylene	12,12	SP-4
Vinyl Radical	11,11	SP-4
Ethane	14,14	SP-6
Ethyl Radical	13,13	SP-6
Pericyclic Reaction 1	4,4	SP-2
Pericyclic Reaction 2	6,6	SP-3
Pericyclic Reaction 3	8,8	SP-4
Pericyclic Reaction 4	4,4	SP-2
Pericyclic Reaction 5	4,4	SP-2
Twisted and planar ethylene	12 electrons in 12 orbitals	2 electrons in 1 orbital (SP-1)
1,4-didehydrobenzene	8 electrons in 8 orbitals	8 electrons in 8 orbitals divided into four spaces (SP-4)
α,3-didehydrotoluene	8 electrons in 8 orbitals	8 electrons in 8 orbitals divided into four spaces (SP-4)

This journal is © The Royal Society of Chemistry 2015

Please do not adjust margins

Please do not adjust margins

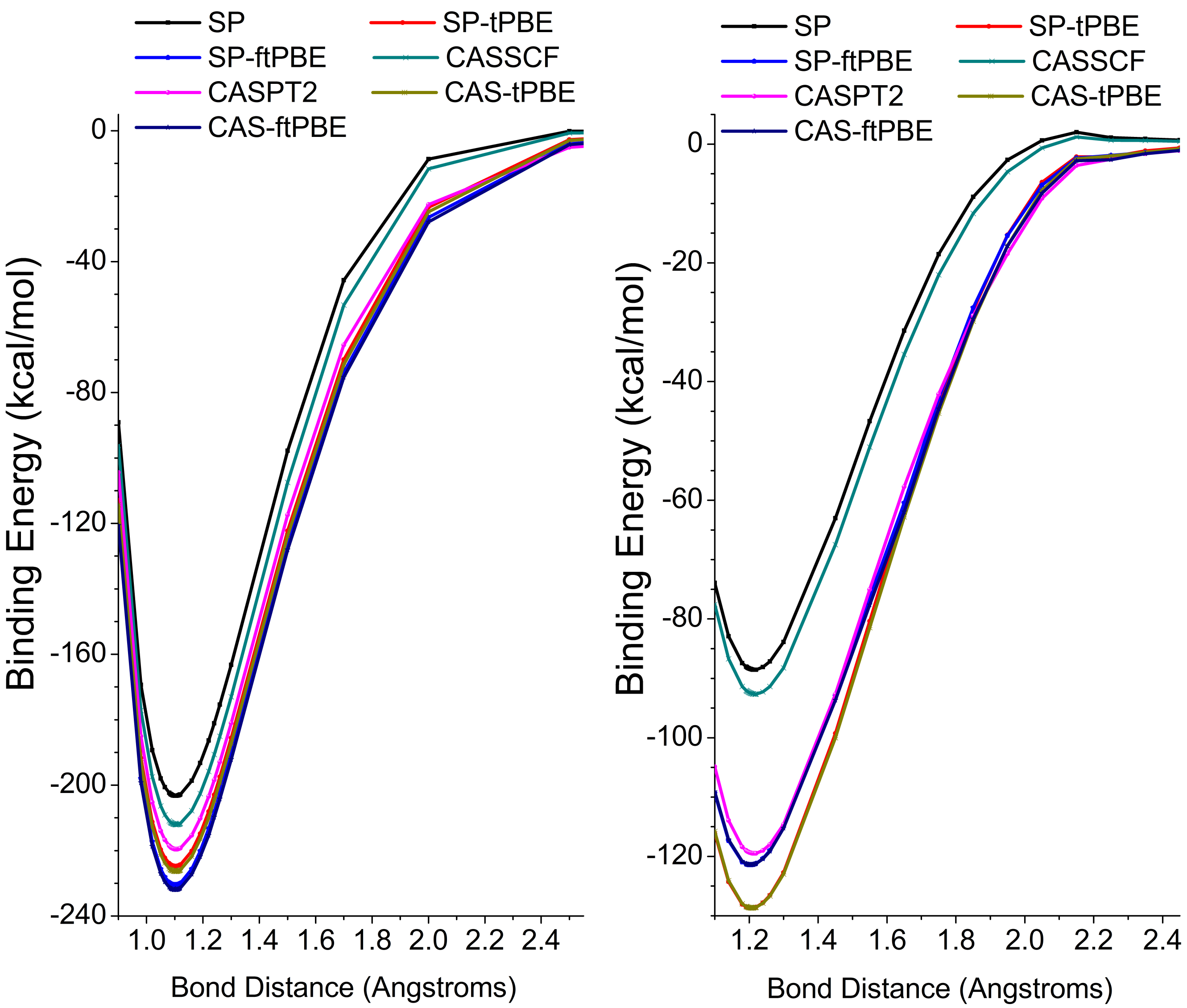

Figure SI1: Calculated potential energy curves of N_2 (*left*) and O_2 (*right*). The binding energies (in kcal/mol) are reported as a function of bond distance, with zero set at 12 Å. At many internuclear distances, the curves obtained with SP-tPBE overlap with those obtained with CAS-tPBE. Similarly the SP-ftPBE and CAS-ftPBE curves overlap.

Please do not adjust margins
